# Mechanistic insights into miR-4775-mediated regulation of pancreatic cancer cell invasion and migration through BRMS1L

**DOI:** 10.1186/s41065-025-00619-w

**Published:** 2026-01-05

**Authors:** Yaxi Song, Bing Han, Qianli Liu, Rui Fan, Xiao Yang

**Affiliations:** 1 The First Department of Oncology, Xinxiang Central Hospital, The Fourth Clinical College of Xinxiang Medical University, Xinxiang, 453000 China; 2 Gastroenterology Department, Peking University Third Hospital Qinhuangdao Hospital, Qinhuangdao, 066000 China; 3 Jiamusi University, Jiamusi, 154007 China; 4 Department of Anus and Intestines, Harbin Traditional Chinese Medicine Hospital, Harbin, 150000 China; 5 Digestive Oncology Department, Changzhou Third People’s Hospital, No. 300, Lanling North Road, Changzhou, 213000 China

**Keywords:** Pancreatic cancer, MiR-4775, Breast cancer metastasis suppressor 1-like, Prognosis

## Abstract

**Background and aims:**

This study aims to investigate the effects of miR-4775 on pancreatic cancer cell (PC) invasion and migration, and to elucidate the underlying molecular mechanisms.

**Methods:**

Quantitative Real-Time Reverse Transcription (RT-qPCR) was performed to analyze miR-4775 and BRMS1L expression levels in both human PC tissues and human pancreatic carcinoma cells‌ (PANC-1) lines. The prognostic value of miR-4775 in PC patients was evaluated through survival analysis. The CCK-8 assay was employed to assess cell viability, while Transwell assays were utilized to evaluate invasion and migration capabilities. The regulatory interaction between miR-4775 and BRMS1L was confirmed by dual-luciferase reporter assay.

**Results:**

Comparative analysis revealed significantly elevated miR-4775 expression in PC tissues versus adjacent normal tissues, with high miR-4775 expression correlating with poorer patient prognosis. Functional studies demonstrated that inhibition of miR-4775 significantly attenuated cellular viability, migration, and invasion capabilities, while knockdown of breast cancer metastasis suppressor 1-like‌ (BRMS1L) effectively rescued these suppressive effects. The luciferase reporter assay confirmed a direct negative regulatory relationship between miR-4775 and its target gene BRMS1L.

**Conclusions:**

These findings demonstrate that miR-4775 regulates PC progression by modulating cancer cell viability, migration, and invasion through BRMS1L-mediated mechanisms.

## Introduction

Pancreatic cancer (PC) is a highly aggressive malignancy affecting the gastrointestinal tract, ranking within the top 12 most prevalent cancers globally [1]. Its etiology involves multifactorial interactions, including individual characteristics (e.g., advanced age, genetic mutations), lifestyle/environmental factors (e.g., trace element exposure), and pre-existing conditions (e.g., chronic pancreatitis) [2]. Clinical manifestations are heterogeneous, with early-stage symptoms often nonspecific. Disease progression typically presents with persistent abdominal pain, jaundice, gastrointestinal dysfunction, and glucose metabolism abnormalities. PC exhibits aggressive metastatic behavior and high recurrence rates, compounded by poor prognosis [3]. Due to the absence of pathognomonic early signs and limited diagnostic tools, approximately 90% of cases are diagnosed at advanced stages, with a 5-year relative survival rate of merely 10% [4]. Current therapeutic options are further constrained by drug resistance.

MicroRNAs (miRNAs) are short non-coding RNA molecules involved in regulating cell cycle progression, stress responses, and post-transcriptional gene regulation. They play critical roles in cancer initiation, growth, and metastasis by interacting with nearly all intracellular signaling pathways, thereby influencing tumorigenesis and progression [5]. MiRNAs serve as prognostic biomarkers, with studies indicating that elevated miR-27a-3p and reduced miR-132-3p expression predict poor outcomes in breast cancer and gastric cancer, respectively [6]. These molecules exert gene regulatory functions by binding to non-coding regions within the 3’UTR of target mRNAs, leading to translational repression or mRNA degradation [7]. Studies implicate miR-4775 in esophageal squamous cell carcinoma progression and chemoresistance [8], while its elevated expression correlates with metastasis, recurrence, and enhanced invasiveness, migration, and epithelial-mesenchymal transition in colorectal cancer cells [9]. The breast cancer metastasis suppressor 1-like (BRMS1L) protein, initially identified as a component of histone deacetylase (HDAC) complexes, suppresses target gene transcription [10]. Research demonstrates that miR-934 promotes ovarian cancer cell proliferation by directly targeting BRMS1L [11]. Additionally, BRMS1L inhibits breast cancer cell migration and invasion through suppression of epithelial-mesenchymal transition [12], restricts invasion and metastasis in cervical squamous cell carcinoma [13], and serves as a survival predictor in glioblastoma [14].

Our preliminary findings detected elevated miR-4775 expression in PC tissues, suggesting its potential role as a tumor-promoting factor in PC pathogenesis. This study, therefore, aims to elucidate the functional mechanisms of miR-4775 in PC at the cellular level.

## Materials and methods

### Clinical data and specimen collection

Clinical records and electronic pathology data were systematically reviewed to retrieve patient demographics. Paired tumor and paracancer tissues (≥ 2 cm from the tumor margin, confirmed pathologically to be free of tumor infiltration) were collected from 140 PC patients who underwent surgical resection at Harbin Traditional Chinese Medicine Hospital between January 2021 and June 2022. All specimens were snap-frozen in liquid nitrogen. Inclusion criteria comprised: (1) histologically confirmed pancreatic adenocarcinoma, and (2) age > 18 years. Exclusion criteria were: (1) prior radiotherapy or chemotherapy, (2) coexisting primary malignancies, or (3) concurrent cardiac/hepatic/renal failure. The cohort consisted of 73 males and 67 females (mean age: 62.37 ± 7.26 years).

All participants provided written informed consent for sample collection and research utilization, and the study was approved by the Ethics Committee of Harbin Traditional Chinese Medicine Hospital (approval number: No. 2020167).

### Bioinformatics

This study analyzed gene expression profiles from the GEO database GSE163031 (https://www.ncbi.nlm.nih.gov/geo), comparing pancreatic ductal adenocarcinoma tissues with non-cancerous pancreatic tissue samples. Potential downstream mRNA targets of miR-4775 were subsequently predicted using the TargetScanHuman platform (https://www.targetscan.org/), MicroT-CDS (http://diana.imis.athena-innovation.gr/DianaTools/index.php), and mirDIP (https://ophid.utoronto.ca/mirDIP/). Additionally, by querying the GeneCsrds (https://www.genecards.org/) with ‘pancreatic cancer’ as the disease keyword, a list of candidate genes associated with this disease was obtained.

### RT-qPCR assay

Total RNA purification from tissue and cell samples was performed using RNAiso Plus (Takara Bio, Japan). RNA extraction, the quality and quantity of RNA (A260/280 = 1.8–2.0.8.0, concentration: Concentration > 0.2 µg/µL, RIN > 7) were assessed by Genova Nano spectrophotometer (Jenway, UK) and 2100 Bioanalyzer (Agilent Technologies). Reverse transcription was performed using the High-Capacity cDNA Reverse Transcription Kit (Thermo Fisher Scientific, USA). Quantitative PCR was performed using the MiniOpticon Real-Time PCR System (Bio-Rad, USA) with iQ SYBR Green SuperMix (Bio-Rad, USA), following the manufacturer’s protocols, and calculated using the 2^−ΔΔCt^ method. The amplification reaction conditions were as follows: initial denaturation at 94 °C for 5 min, followed by 30 cycles of denaturation at 94 °C for 30 s, annealing at 58 °C for 45 s, extension at 72 °C for 30 s, and a final extension at 72 °C for 10 min [15].

The expression level of miR-4775 was measured in a 15 µL reaction volume employing the SYBR Premix Ex Taq™ kit (Takara, Japan) with specific primers (forward: 5ʹ-GCGCGTTAATTTTTTGTTTCG-3ʹ; reverse: 5ʹ-AGTGCAGGGTCCGAGGTATT-3ʹ). U6 (forward: 5ʹ-GCTTCGGCAGCACATATACT-3ʹ; reverse: 5ʹ-AACGCTTCACGAATTTGCGT-3ʹ) small nuclear RNA served as the normalization control. BRMS1L (forward: 5ʹ-GAGCGGTTGAGTCAGGTGG-3ʹ; reverse: 5ʹ-CCTTTGTGCGAATCTGCATGT-3ʹ). GAPDH (forward: 5ʹ-GATGCTGGCGCTGAGTACG-3ʹ; reverse: 5ʹ-GCTAAGCAGTTGGTGGTGC-3ʹ) as the normalization control.

### Prognostic value analysis‌

Based on the mean miR-4775 concentration (expression of miR-4775 = 2.42) in PC tissues serving as the stratification threshold, patients were categorized into “high miR-4775” (miR-4775 expression level ≥ 2.42) and “low miR-4775” (miR-4775 expression level< 2.42) groups with subsequent analysis of survival outcomes.

### Cell culture and treatment

PANC-1 (National Cell Centre, China) cells were cultured in DMEM (Gibco, USA) supplemented with 10% fetal bovine serum (FBS; Gibco, USA) and 1% penicillin/streptomycin (Gibco, USA) at 37 °C under 5% CO₂ in a humidified incubator.

### Transfection

24 h prior to transfection, logarithmically growing PANC-1 cells were seeded in 6-well plates at a density of 5 × 10⁵ cells per well. Upon reaching approximately 80% confluency, the culture medium was replaced with serum- and antibiotic-free Opti-MEM (Gibco, USA). Transfection was performed according to the manufacturer’s protocol for Lipofectamine™ 2000 (Invitrogen, USA), introducing either inhibitor NC, miR-4775 inhibitor, si-NC, or si-BRMS1L into the cells. A final concentration of either the inhibitor NC, miR-4775 inhibitor (100nM), si-NC or si-BRMS1L(50nM) was transfected into the cells using Lipofectamine™ 2000 (Invitrogen) according to the manufacturer’s protocol. Briefly, siRNA or miR-4775 inhibitor was mixed with 1 µL lipofectamine 2000, the transfected cells were maintained at 37 °C in a humidified 5% CO₂ incubator. After 4 h of incubation, the medium was replaced with complete growth medium, followed by an additional 48-hour culture period before subsequent experimental procedures.

### Cell viability activity assay

For cell viability analysis, PANC-1 cells were seeded in 24-well plates at a density of 1 × 10⁴ cells/well and cultured overnight. Following incubation, 100 µL of CCK-8 reagent (Fdbio Science, China) was added to each well. Cells were further incubated for 2 h, protected from light exposure. ‌Absorbance measurements at 450 nm were performed using a BioTek microplate reader to quantify cellular viability activity, with a reference wavelength of 650 nm applied for background correction.

### ‌‌Cell migration and invasion

‌To evaluate cellular invasiveness, PANC-1 cells were serum-starved for 24 h prior to experimentation. Subsequently, 5 × 10⁴ cells/well were suspended in 200 µL of serum-depleted medium and transferred to the upper chamber of transwell inserts pre-coated with Matrigel matrix (diluted 1:8 in serum-free DMEM and allowed to polymerize at 37 °C for 3 h) and incubated for 24 h. ‌For migration assays‌, cells (2 × 10⁴ cells/well) were seeded into uncoated chambers and incubated for 24 h. ‌All assays used‌ 500 µL complete medium with 10% FBS as a chemoattractant in the lower chambers. ‌After incubation‌, cells on the membrane undersurface were fixed in 4% paraformaldehyde, stained with 0.1% crystal violet, and imaged by light microscopy. Invasive/migratory cells were quantified by counting five random fields per membrane.

### Western blot

Cells were washed three times with PBS and lysed on ice for 30 min in RIPA buffer (Solarbio, China) supplemented with 1% protease/phosphatase inhibitor cocktail PMSF (Solarbio, China). Total protein extracts were separated by 10% SDS-PAGE, transferred to PVDF membranes (Millipore, Bedford, MA, USA), and incubated overnight at 4 °C with primary antibodies against β-actin (1:10,000, #14395-1-AP, Proteintech) and BRMS1L (1:2000, ab155188, Abcam). Protein blots were imaged using the Odyssey infrared imaging system (Li-COR Biosciences, Nebraska, USA).

### Dual-luciferase reporter assay

Bioinformatic prediction of miR-4775-BRMS1L targeting interactions was performed using TargetScan Human, followed by construction of BRMS1L wild-type (BRMS1L-WT) and mutant (BRMS1L-Mut) reporter vectors in the pmirGLO dual-luciferase reporter vector (Promega, USA). Log-phase cells seeded in 24-well plates were co-transfected using Lipofectamine™ 2000 (Invitrogen, USA) with either BRMS1L-WT or BRMS1L-MUT alongside miRNA negative control (miR NC), miR-4775 mimics, or miR-4775 inhibitor. PANC-1 were seeded in 24-well plates, and co-transfected with miR-4775 mimic or miR-4775 inhibitor‌ (100nM) and wild-type (BRMS1L-wt) or mutant (BRMS1L-mut) 3’-UTR‌ (0.5 µg) by using Lipofectamine 2000(1.5 µL). Relative luciferase activity was quantified after 48-hour incubation.

### Statistical analysis

Statistical analyses were performed using SPSS 25.0 (USA), while graphical representations were generated with GraphPad Prism 10.1.2 (USA), employing Student’s t-test and chi-square tests for intergroup comparisons, data were checked for normality via the Kolmogorov-Smirnov (K-S) normality test (sample size exceeding 50), one-way and multivariate ANOVA for multigroup analyses, and Kaplan-Meier methodology with log-rank testing to evaluate miR-4775 expression’s association with survival outcomes.

## Results

### Expression of miR-4775

**Fig. 1:**
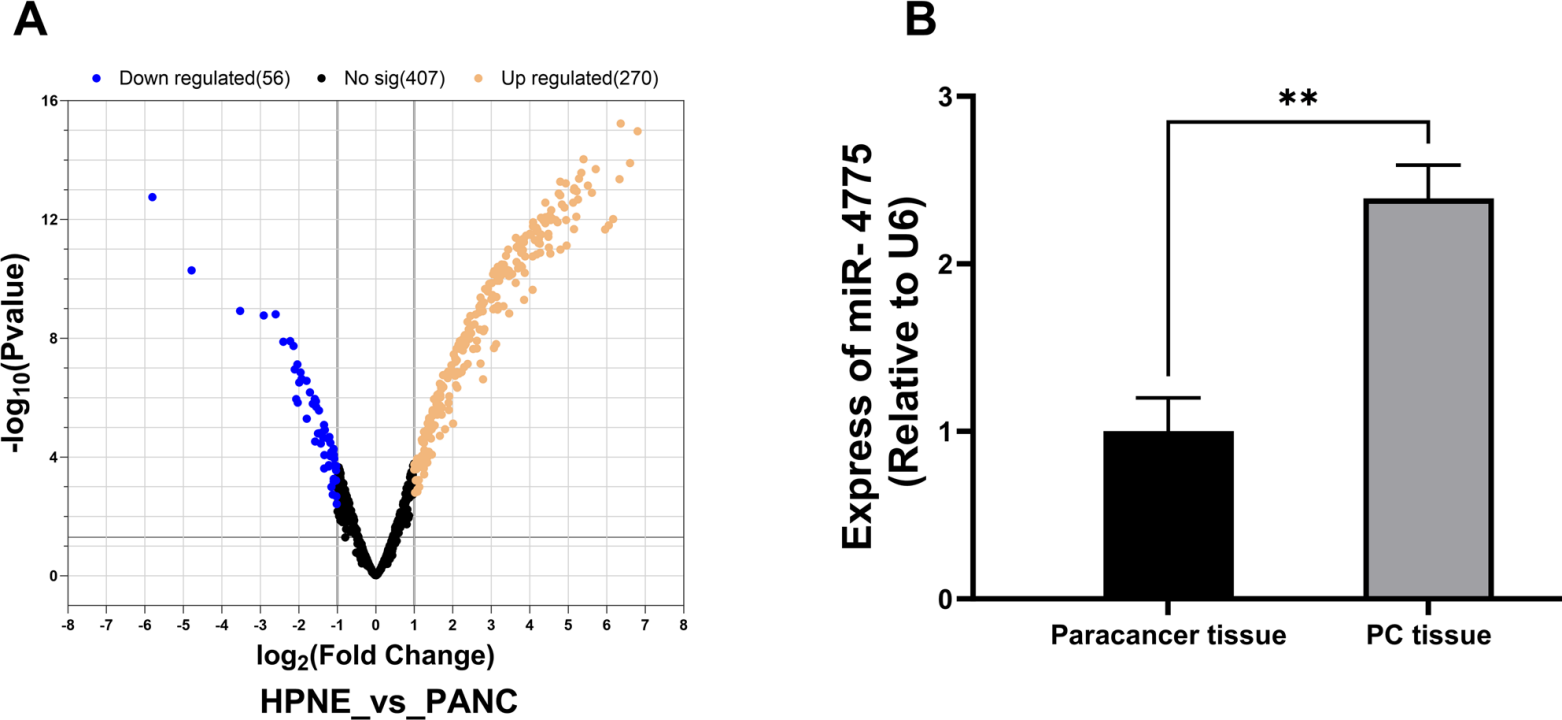
Expression levels of miR-4775 in tissues. **A** Volcano plot of differential miRNA expression. **B** Expression levels of miR-4775 in tissues

‌‌Based on volcano plot analysis demonstrating that miR-4775 exhibited the log2-fold change among differentially expressed circRNAs in PANC-1 cells, this microRNA was consequently selected for further mechanistic investigation (Fig. 1A). RT-qPCR analysis demonstrated significantly elevated miR-4775 expression in PC tissues compared to paracancer tissues (*P* < 0.01) (Fig. 1B).

### ‌miR-4775 associates with poor prognosis in PC patients

**Fig. 2:**
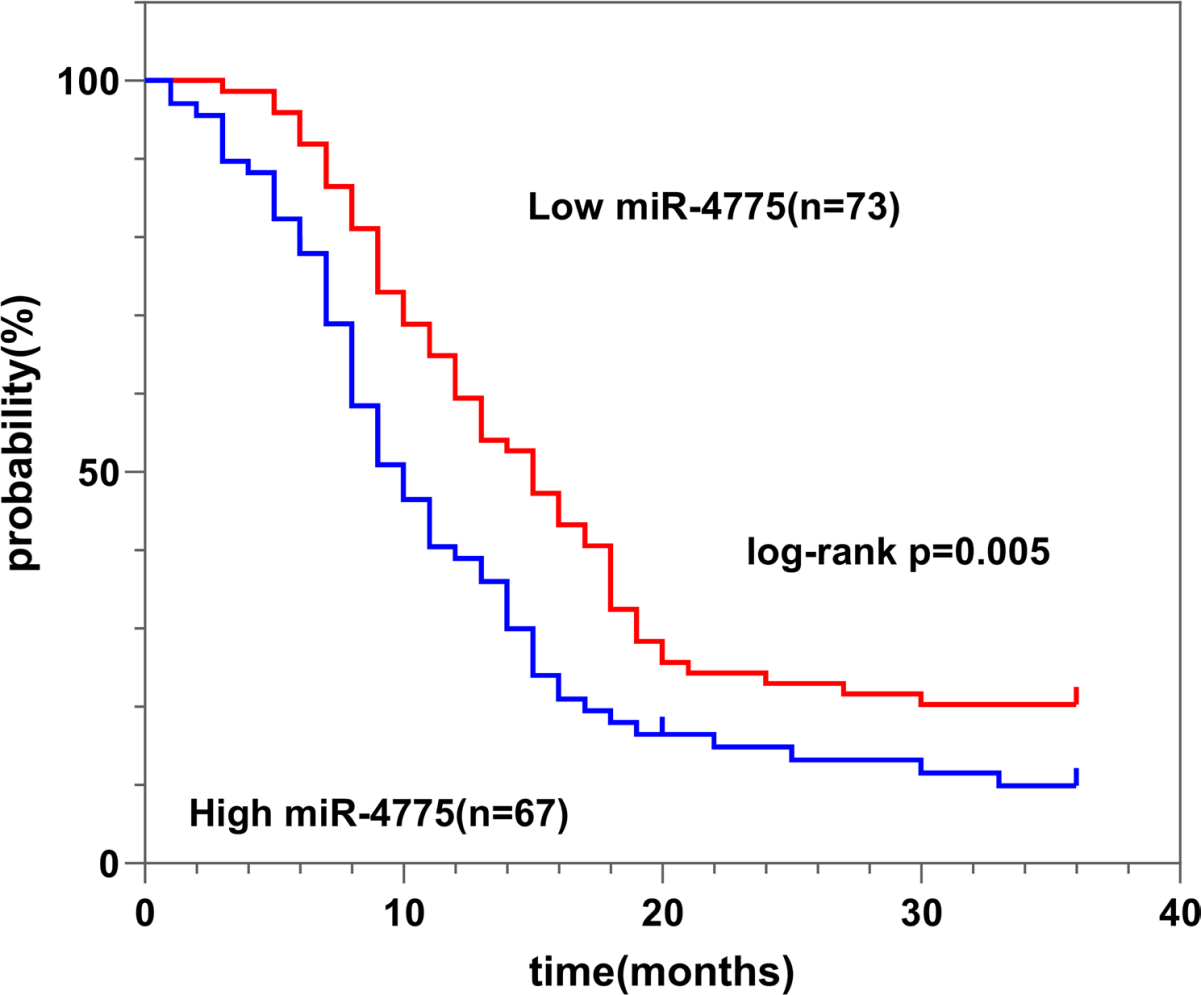
Kaplan-Meier curve based on miR-4775 expression levels

‌Patient baseline characteristics are summarized in Table 1. Univariate Cox proportional hazards regression analysis revealed no significant association between PC patient survival and gender, age, or tumor location. However, significant correlations were observed with lymph node metastasis status, AJCC stage, and miR-4775 expression levels (*P* < 0.05) (Table 2). Furthermore, Kaplan-Meier analysis demonstrated that patients with high miR-4775 expression in tumor tissues exhibited poorer prognosis compared to those with low expression (log-rank *p* = 0.005) (Fig. 2). These findings suggest miR-4775 possesses independent prognostic significance, with elevated expression indicating an unfavorable prognosis in PC patients.

**Table 1:** Comparison of baseline characteristics

	cases	miR-4775		χ2	*P*-value
		Low express	High express		
Gender				0.273	0.601
male	72	36	36		
female	68	37	31		
Age				0.587	0.444
≤ 60	59	33	26		
>60	81	40	41		
Tumor Location				0.110	0.740
Pancreatic head	98	52	46		
Tail of pancreas	42	21	21		
Differentiation Grade				1.355	0.508
G1	26	11	15		
G2	59	33	26		
G3	55	29	26		
T Stage				0.367	0.832
T1	27	15	12		
T2	82	41	41		
T3 + T4	31	17	14		
Lymph Node Metastasis				6.599	0.010
no	52	35	17		
yes	88	38	50		
AJCC Staging System				8.468	0.004
I-II A	66	43	23		
II B + III	74	30	44		
Perineural Invasion				2.174	0.140
no	62	28	34		
yes	78	45	33		
Lymphovascular Invasion				0.046	0.831
no	76	39	37		
yes	64	34	30		

**Table 2:** Association of clinical features with the overall survival of patients

	HR factor	95%CI	*p* value
Gender	0.965	0.653–1.425	0.857
Age	1.345	0.896–2.020	0.153
Tumor location	2.458	1.592–3.797	0.06
Lymphatic metastasis	2.636	1.699–4.090	< 0.0001****
AJCC	5.272	3.773–7.367	< 0.0001****
miR-4775	1.487	0.984–2.248	< 0.0001****

****p < 0.0001

### ‌‌miR-4775 promotes migration and invasion

**Fig. 3:**
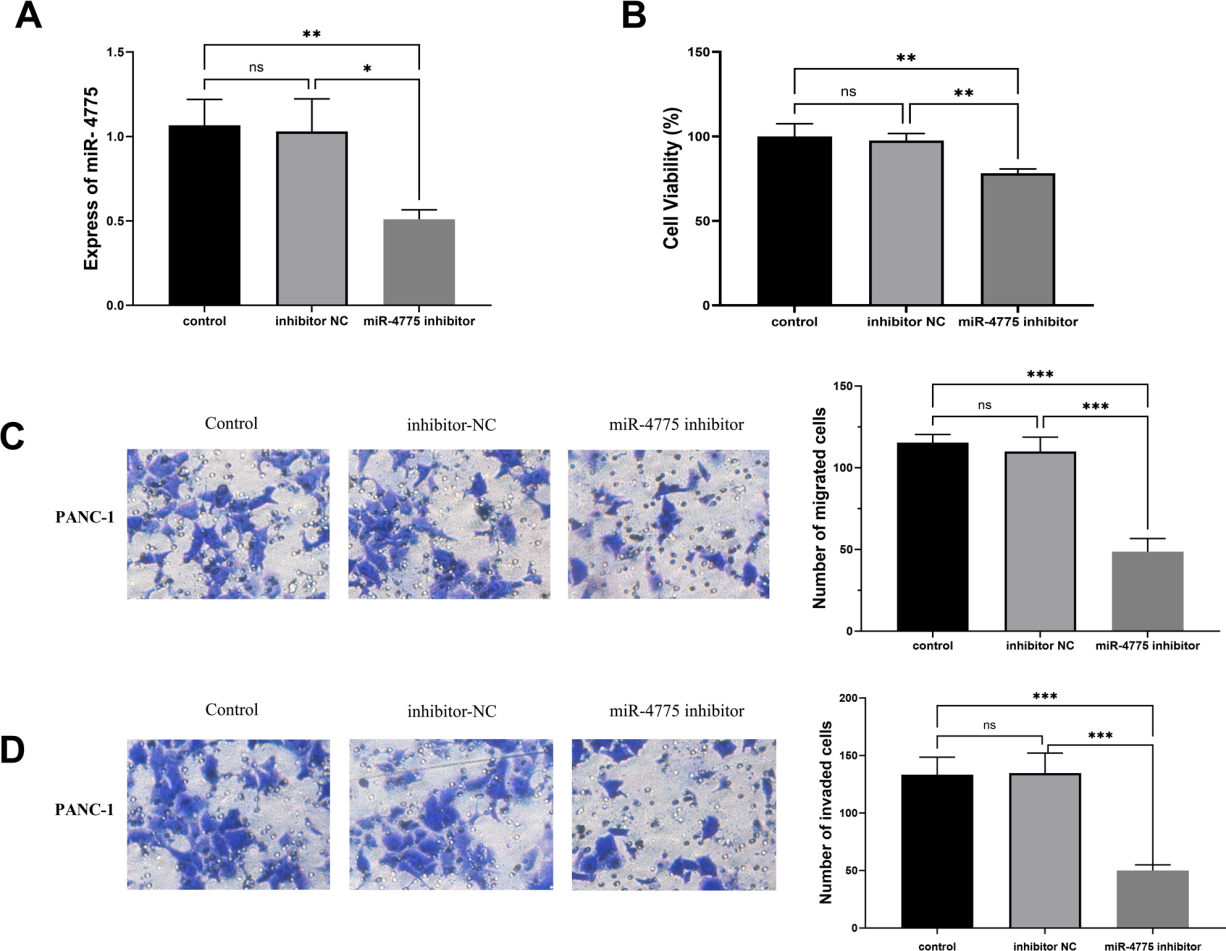
PANC-1 cells were transfected with miRNA-NC, miR-4775 mimics, inhibitor NC, and miR-4775 inhibitor for 48 h. **A** Expression levels of miR-4775. **B** Viability of PANC-1 cells. **C** Number of migrated cells (representative image). **D** Number of invaded cells (representative image)

‌Further investigation into the cellular regulatory impact of miR-4775 demonstrated significantly downregulated miR-4775 expression in the miRNA inhibitor group compared with the inhibitor NC group (*P* < 0.05) (Fig. 3A). Functional analyses revealed that transfection with miRNA inhibitor substantially suppressed cell viability (*P* < 0.01), migration (*P* < 0.001), and invasion (*P* < 0.001) (Figs. 3B-D), indicating a critical association between miR-4775 expression levels and metastatic behaviors in PANC-1 cells.

### miR-4775 directly targets BRMS1L

**Fig. 4:**
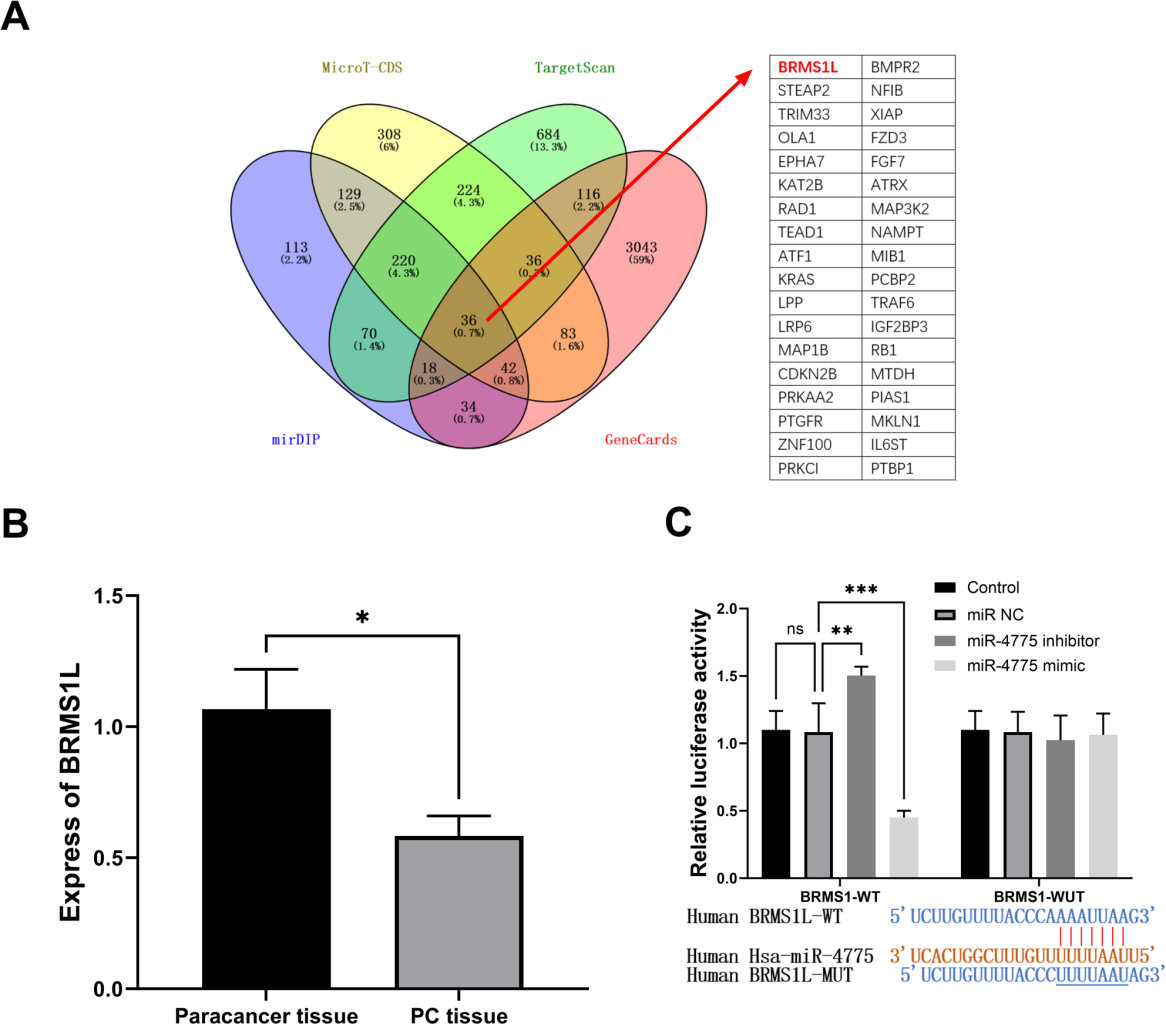
‌Targeting mechanism of miR-4775 on BRMS1L. **A** Venn diagram for the gene target miRNAs of miR-4775. **B** Expression levels of BRMS1L in tissues. **C** Regulatory Interaction between BRMS1L and miR-4775 in a targeted manner

To identify the target genes of miR-4775, we performed bioinformatic analysis using TargetScan 8.0, MicroT-CDS, mirDIP, and GeneCsrds (Fig. 4A). We screened cell viability, invasion, and migration pathway-related genes containing miR-4775 binding sites in their 3′UTRs, as well as genes related to pancreatic cancer. Subsequently, BRMS1L was identified as a candidate gene. Quantitative PCR analysis revealed a statistically significant reduction in BRMS1L expression levels in tumor tissues compared with adjacent non-tumorous tissues (*P* < 0.05) (Fig. 4B). Bioinformatic prediction via Target Scan Human revealed binding sites between miR-4775 and BRMS1L. To investigate their regulatory relationship, dual-luciferase assays in PANC-1 cells showed miR-4775 inhibitor transfection substantially enhanced luciferase activity in wild-type (WT) constructs (*P* < 0.001), whereas miR-4775 mimic significantly suppressed activity (*P* < 0.05), with no effects observed in mutant (MUT) constructs (Fig. 4C), collectively proving miR-4775 specifically and negatively regulates BRMS1L expression.

### miR-4775 modulates the progression of PC through the BRMS1L

**Fig. 5:**
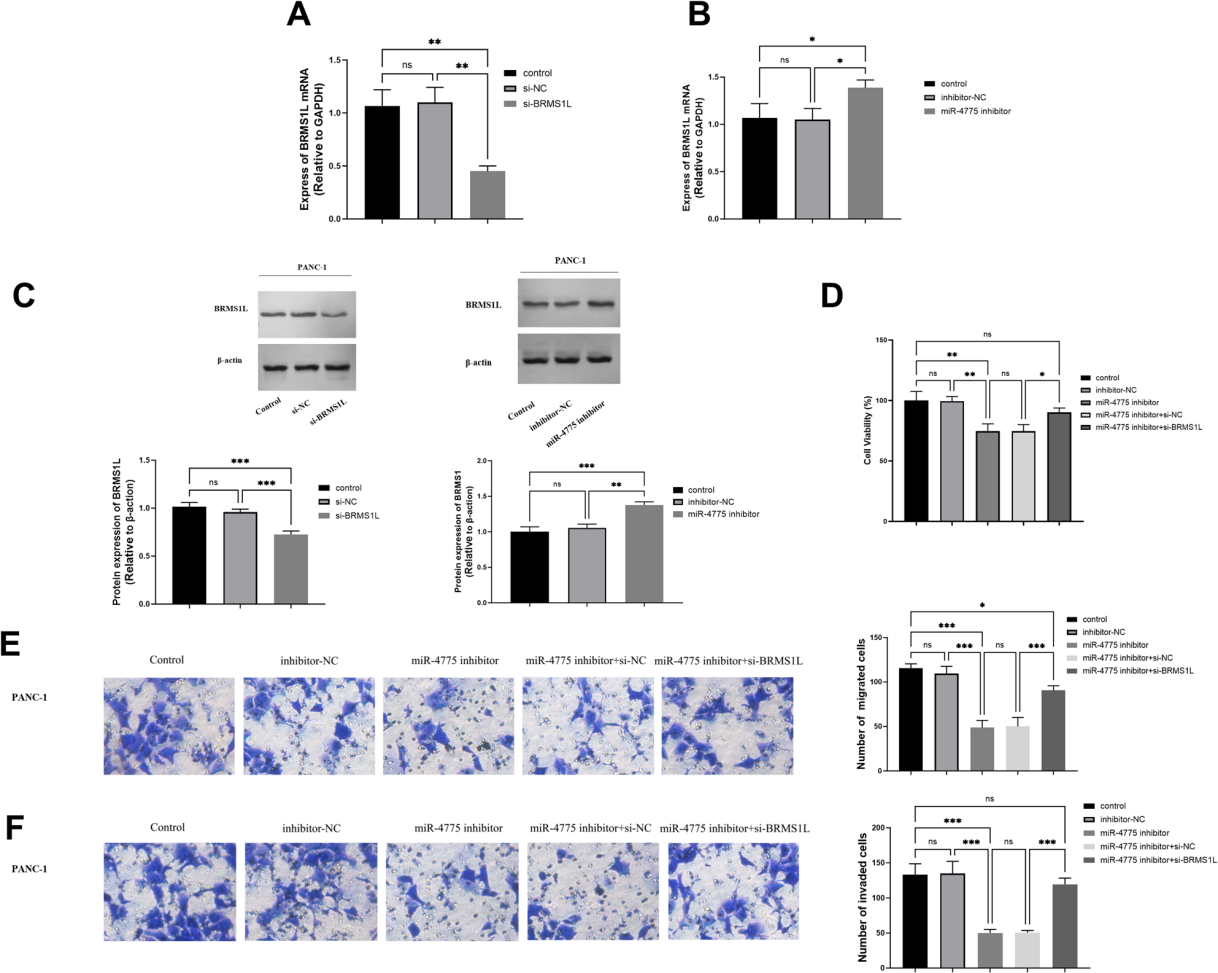
PANC-1 cells were transfected with si-NC, si-BRMS1L, miR inhibitor+si-NC, and miR inhibitor+si-BRMS1L for 48 h. **A** Expression levels of BRMS1L after transfection with si-BRMS1L. **B** Expression levels of BRMS1L after transfection with miR-4775 inhibitor. **C** Protein expression of BRMS1L was examined by Western blot. **D** Cell viability of PANC-1 cells. **E** Number of migrated cells (representative image). **F** Number of invaded cells (representative image)

To elucidate miR-4775-mediated regulatory mechanisms targeting BRMS1L, we examined BRMS1L’s functional impact. Cellular analysis revealed significant BRMS1L downregulation in si-BRMS1L versus si-NC groups (*P* < 0.01) (Fig. 5A). Additionally, the miR-4775 inhibitor group exhibited increased BRMS1L expression relative to the inhibitor-NC group (*P* < 0.05) (Fig. 5B). Western blot analysis further confirmed that BRMS1L protein levels were reduced in the si-BRMS1L group compared to both the control (*P* < 0.001)and si-NC groups, while the miR-4775 inhibitor group showed elevated BRMS1L protein expression (*P* < 0.01) (Fig. 5C).

‌Functional analyses demonstrated that co-transfection with miR-inhibitor and si-BRMS1L significantly enhanced viability (*P* < 0.05), migration (*P* < 0.001), and invasion (*P* < 0.001) capacities of PANC-1 cells relative to miR-inhibitor plus non-targeting siRNA controls (Fig. 5D-F), collectively indicating that BRMS1L knockdown functionally rescues the suppressive effects of miR-4775 inhibition, establishing BRMS1L as a critical downstream mediator of miR-4775 in modulating PC metastasis.

## Discussion

The pancreas, the second-largest digestive gland in humans, performs both exocrine and endocrine functions. Its exocrine component primarily consists of acinar cells and ducts that secrete digestive enzymes into the intestinal tract, while the endocrine portion, known as pancreatic islets, is diffusely distributed throughout the parenchyma and synthesizes various hormones, including insulin and glucagon, to regulate glucose metabolism. PC is widely recognized for its aggressive nature and poor prognosis [16], exhibiting the highest incidence and mortality rates among gastrointestinal malignancies, with the lowest survival rates. Due to its deep anatomical location and the lack of early diagnostic methods, 70–80% of patients present with locally advanced or metastatic disease at diagnosis, with only 10% being candidates for surgical resection [17]. Pancreatic ductal adenocarcinoma (PDAC), originating from the epithelial cells of pancreatic ducts, accounts for over 90% of PC cases and is characterized by malignant proliferation with invasive growth patterns.

As a member of the microRNA family, miR-4775 is increasingly implicated in the progression of multiple human malignancies. It is widely acknowledged that malignant progression and dissemination constitute primary drivers of recurrence [18]. Beyond its role in colorectal cancer, where miR-4775 facilitates cancer cell invasion and metastasis by mediating epithelial-mesenchymal transition [9], this microRNA also accelerates breast cancer progression [20] and enhances cellular migration and invasion in lung cancer [15]. As a BRMS1 analog, BRMS1L exerts its functions through multiple signaling pathways, predominantly by enhancing histone deacetylase complex activity to recruit mSin3A-HDAC complexes. This modulates tumor growth-related promoters, thereby suppressing viability, invasion, and metastasis in diverse cancers, including lung and breast carcinomas [19]. Furthermore, BRMS1L maintains transcriptional repression of Wnt pathway target genes [10], functioning as a tumor suppressor. It critically regulates malignant behaviors such as invasion and metastasis, decisively influencing epithelial-mesenchymal transition in tumor cells. Studies demonstrate that miR-93-5p promotes lacrimal adenoid cystic carcinoma pathogenesis by targeting BRMS1L [20], while miR-17-5p modulates nasopharyngeal carcinoma cell invasion and migration through BRMS1L targeting. Additionally, the miR-96/−182/−183 cluster promotes epithelial-mesenchymal transition and subsequent invasion in breast cancer [21].

This study identified elevated miR-4775 expression in PC tissues, where its overexpression promoted PANC-1 cell viability, invasion, and migration, whereas BRMS1L overexpression suppressed these malignant behaviors. Dual-luciferase reporter assays validated the negative regulation of BRMS1L by miR-4775, therefore demonstrating that miR-4775 promotes malignant phenotypes in PC PANC-1 cells through targeted downregulation of BRMS1L expression.

## Conclusions

miR-4775 modulates PC cell viability, migration, and invasion through BRMS1L regulation, thereby influencing PC progression.

## Data Availability

The datasets used and/or analysed during the current study are available from the corresponding author on reasonable request.
